# Epidemiology of Rotavirus and Cholera in Children Aged Less Than Five Years in Rural Bangladesh

**DOI:** 10.3329/jhpn.v29i1.7560

**Published:** 2011-02

**Authors:** A.K. Siddique, Sirajuddin Ahmed, Anwarul Iqbal, Arif Sobhan, Goutam Poddar, Tasnim Azim, D.A. Sack, Mustafizur Rahman, R.B. Sack

**Affiliations:** ^1^ ICDDR, B, GPO Box 128, Dhaka 1000, Bangladesh; ^2^ Johns Hopkins Bloomberg School of Public Health, 615 North Wolfe Street, Baltimore, MD 21205, USA

**Keywords:** Cholera, Diarrhoea, Hospitalizations, Rotavirus infections, *Vibrio cholerae*, Bangladesh

## Abstract

Despite the known presence of rotavirus-associated diarrhoea in Bangladesh, its prevalence, including records of hospitalization in rural health facilities, is largely unknown. In a systematic surveillance undertaken in two government-run rural health facilities, 457 children, aged less than five years, having acute watery diarrhoea, were studied between August 2005 and July 2007 to determine the prevalence of rotavirus. Due to limited financial support, the surveillance of rotavirus was included as an addendum to an ongoing study for cholera in the same area. Rotavirus infection was detected in 114 (25%) and *Vibrio cholerae* in 63 (14%) children. Neither rotavirus nor *V. cholerae* was detected in 280 (61%) samples; these were termed ‘non-rotavirus and non-cholera’ diarrhoea. Both rotavirus and cholera were detected in all groups of patients (<5 years). The highest proportion (41%; 47/114) of rotavirus was in the age-group of 6-11 months. In children aged less than 18 months, the proportion (67%; 76/114) of rotavirus was significantly (p<0.001) higher than that of cholera (16%; 10/63). By contrast, the proportion (84%; 53/63) of cholera was significantly (p<0.001) higher than that of rotavirus (33%; 38/114) in the age-group of 18-59 months. During the study period, 528 children were hospitalized for various illnesses. Thirty-eight percent (202/528) of the hospitalizations were due to acute watery diarrhoea, and 62% were due to non-diarrhoeal illnesses. Rotavirus accounted for 34% of hospitalizations due to diarrhoea. Severe dehydration was detected in 16% (74/457) of the children. The proportion (51%; 32/63) of severe dehydration among *V. cholerae*-infected children was significantly higher (p<0.001) compared to the proportion (16%; 18/114) of rotavirus-infected children. The study revealed that 12-14% of the hospitalizations in rural Bangladesh in this age-group were due to rotavirus infection, which has not been previously documented.

## INTRODUCTION

Rotavirus is the most common cause of diarrhoea in infants and young children in both developed and developing countries (1). On a global scale, rotavirus is responsible for nearly 140 million cases of diarrhoea each year in children aged six months to two years (2, 3). It is also one of the major contributors to deaths of infants and young children in developing countries (2), claiming 500, 000-600, 000 lives each year (4–6), and up to 85% of these deaths occur in low-income countries (4). Diarrhoea due to rotavirus in children is recognized as the leading cause of hospitalizations worldwide (7).

The disease affects infants and children aged three months and by age 3-4 years; virtually all children have had the disease. Previous infection offers protection from subsequent illnesses. However, re-infections are frequent but subsequent illnesses tend to be less severe than the first infection (8). Many rotavirus infections are asymptomatic, particularly among infants aged less than three months, older children, and adults (9, 10). Immunity from repeated exposures to rotavirus probably accounts for the high infection-to-illness ratio among older children and adults. Rotavirus infections are likely to be associated with dehydration (11, 12). The majority of deaths from diarrhoea due to rotavirus occur in Africa, the Indian Sub-continent, and Latin Americas. Despite the widespread recognizable prevalence of diarrhoea due to rotavirus in Asia, including the Indian Sub-continent and the Asia-Pacific region, information on the incidence and the disease is grossly deficient (13, 14). Hospitalization data seem to be scantier than incidence data.

Diarrhoeal disease is one of the major public-health problems in Bangladesh. For nearly two decades, it has been recognized that rotavirus is one of the common causes of diarrhoea in children in urban Bangladesh. A surveillance conducted in the late nineties at the ICDDR, B hospital in urban Dhaka suggested that 20% of children seeking treatment in this diarrhoea-treatment facility suffer from rotavirus infection (15). Little is known about the epidemiology of diarrhoea due to rotavirus in rural Bangladesh where about 90% of the population lives. We also do not know much about the magnitude of hospitalization due to rotavirus, particularly in rural health facilities. Results of a recent study in Matlab, where the International Centre for Diarrhoeal Disease Research, Bangladesh (ICDDR, B) maintains a Health and Demographic Surveillance System and a diarrhoea-treatment centre, showed that rotavirus was a major cause of hospitalizations in this treatment centre (16).

This paper presents results of the two-year clinical surveillance of rotavirus-associated diarrhoea involving two rural areas of Bangladesh. The surveillance was undertaken to study the epidemiology of diarrhoea due to rotavirus among children aged less than five years (under-five children) and to assess the rate of hospitalizations due to rotavirus-associa-ted diarrhoea at rural health facilities in Bangladesh. The health facilities where the surveillance of rotavirus was conducted were also the sites for an ongoing epidemiological and ecological study for cholera supported by a separate grant. Adding the specific requirements for the rotavirus surveillance study as an addendum to the cholera protocol rendered the rotavirus study feasible within the limits of financial support offered for the purpose.

## MATERIALS AND METHODS

### Clinical surveillance

The clinical surveillance was conducted in Bakerganj and Mathbaria upazilas (subdistricts), each with a population of ~250,000. Bakerganj is located in the southern region of Bangladesh, 300 km from the capital city Dhaka. Mathbaria is situated in the southern-most region of the country, close to mangrove swamps of the Sunderbans, at a distance of over 350 km from Dhaka city. The facility-based clinical surveillance was conducted in Bakerganj and Mathbaria Upazila Health Complexes (UHCs) which are grassroots-level rural hospitals run by the national health services of the country. Each hospital has 31-bed inpatient-care facility. These hospitals also offer outpatient care and other preventive care services. The UHC is also the referral centre for a number of grassroots-level community clinics.

The method of this surveillance system has previously been described (17). In short, the clinical surveillance was conducted every 15 days between August 2005 and July 2007. A study physician who visited the surveillance site stayed there for three days during which time he personally observed all patients presenting with acute watery diarrhoea. The study physician administered a pre-coded questionnaire and recorded demographic information, history of the onset of illness, and clinical findings. A detailed physical examination for each patient was carried out. The dehydration status of patients was determined objectively using the criteria of the World Health Organization (WHO) and was cate-gorized as ‘no detectable dehydration’, ‘some dehydration’, or ‘severe dehydration’ (18). Standard therapy (rehydration, nutritional support, and antibiotics when necessary) for diarrhoeal illness was given. During the three-day surveillance period, the study physician also collected clinical parameters of all under-five children admitted for inpatient care by the hospital physicians for all types of illness, including acute watery diarrhoea.

A case of rotavirus-associated diarrhoea was defined as an under-five child seeking treatment for diarrhoea at the outpatient or hospitalized in whose stool the presence of rotavirus was demonstrated by means of enzyme immunoassay (19). In this study, no attempt was made to identify pathogens other than rotavirus and cholera associated with watery diarrhoea.

For comparison, this report also includes data of patients infected with *V. cholerae* to determine the severity of dehydration and impact on hospitalization due to rotavirus infection in under-five children.

Data were analyzed using the Epi Info software (version 3.3.2), and the χ^2^-test for difference was used in this study.

### Laboratory methods

Stool specimens were obtained from each patient seen by the study physician for laboratory identification of rotavirus and placed in a sterile screw-cap container. Rectal swabs, collected from all patients, were placed in the Cary-Blair medium for culture of *V. cholerae.* All the specimens were properly labelled with information, including a unique identification number and the date of collection. Specimens were stored temporarily in refrigerators at 4-8 °C before transporting to the ICDDR, B laboratory in Dhaka in cold-boxes with ice-packs.

At the ICDDR, B laboratory, rotavirus antigens (group A rotavirus-specific VP6 proteins) were detected in stool specimens using a solid-phase sandwich-type enzyme immunoassay modelled after the Dakopatts commercial kit (Dakopatts, Copenhagen, Denmark), incorporating rabbit hyperimmune antisera produced at ICDDR, B and an anti-human rotavirus horseradish conjugate (2). A multiplex reverse transcriptase-polymerase chain reaction (RT-PCR) was performed on rotavirus-positive stool samples for rotavirus G and P genotyping using type-specific oligonucleotide primers (2). For the identification of *V. cholerae,* all the rectal swabs were cultured using standard bacteriological methods (20). Swabs were cultured both directly and after six-hour enrichment in alkaline peptone water on selective media at 37 °C. Suspected colonies resembling *V. cholerae* were agglutinated with antisera specific for *V. cholerae* O1 and *V. cholerae* O139.

### Ethics

Informed consent was obtained from parents accompanying the children. The Research Review Committee and the Ethical Review Committee of ICDDR, B and the Institutional Review Board of the Johns Hopkins Bloomberg School of Public Health approved the research protocol.

## RESULTS

The systematic surveillance was conducted between August 2005 and July 2007 in the two surveillance sites. During the period, 457 under-five children with acute watery diarrhoea from both outpatient and inpatient departments were studied. Laboratory examination revealed that 114 (25%) samples were positive for rotavirus antigen; *V. cholerae* was detected in 63 samples (14%); and in 280 (61%) samples, neither rotavirus nor *V. cholerae* was detected (Table 1). Cases of mixed infection with rotavirus and *V. cholerae* were not observed during the study period. For the purpose of this study, under-five patientswere categorized in five different age-groups. The figure shows the distribution of age-specific illness due to rotavirus-associated diarrhoea, cholera, and ‘non-rotavirus and non-cholera’ diarrhoea. Both rotavirus infection and cholera were detected in all the age-groups. The highest age-group frequency (41%; 47/114) of rotavirus was noted in the age-group of 6-11 months. This was much higher than in cholera and ‘non-rotavirus and non-cholera’ diarrhoea patients in this age-group. Further analysis of the age-specific distribution between rotavirus and cholera in children aged less than 18 months revealed that the proportion (67%; 76/114) of rotavirus infection was significantly (p<0.001) higher than the proportion (16%; 10/63) of cholera infection. By contrasts, in children aged 18-59 months, the proportion (84%; 53/63) of *V. cholerae*-associa-ted infection was significantly (p<0.001) higher than that of rotavirus (33%; 38/114). It was also revealed that 77% of the children were rotavirus- infected by the time they were aged 24 months.

**Table 1.** Distribution of acute watery diarrhoea due to rotavirus and *V. cholerae* and ‘non-rotavirus and non-cholera’ infection in under-five children in Bakerganj and Mathbaria, Bangladesh, August 2005–July 2007

Surveillance site	No. of acute watery diarrhoea cases	No. positive				Non-rotavirus and non-cholera cases	
		Rotavirus		*V. cholerae*			
		No.	%	No.	%	No.	%
Bakerganj	197	45	22.8	37	18.8	115	58.4
Mathbaria	260	69	26.5	26	10.0	165	63.5
Total	457	114	24.9	63	13.8	280	61.3

During the surveillance period, 528 under-five children were hospitalized with various illnesses (Table 2) in the two surveillance hospitals (Bakerganj–256 and Mathbaria–272). The hospitalized cases of under-five children were divided into (a) non-diarrhoea cases (326/528) and (b) acute watery diarrhoea cases (202/528). This was done based on the observation that acute watery diarrhoea was the cause of all hospitalized diarrhoea patients in this age-group. Acute watery diarrhoea accounted for 38% (202/528) of hospital admissions compared to non-diarrhoea patients (62%; 326/528). Further analysis of the hospitalized cases of acute watery diar-rhoea revealed that rotavirus infection accounted for 34% (69/202) of the admitted cases compared to cholera (25%; 50/202). The ‘non-rotavirus and non-cholera’ watery diarrhoea was responsible for 41% (83/202) of all diarrhoeal admissions. The highest proportion (42%) of 69 children hospitalized with rotavirus were aged 6-11 months. It was observed that 13% (average of the two sites) of all hospitalizations in our surveillance sites (12% in Bakerganj and 14% in Mathbaria) were due to rotavirus infection. Cholera was responsible for 9.5% of the total admissions. It was also revealed that 16% of all hospitalizations were due to ‘non-rotavirus and non-cholera’ diarrhoea.

**Fig.**
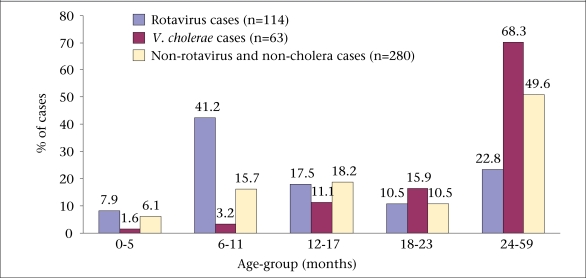
Percent distribution of study children by aetiologic agent and age-group in Bakerganj and Mathbaria, Bangladesh, August 2005–July 2007

**Table 2.** Distribution of hospitalization of under-five children by cause in Bakerganj and Mathbaria: Bangladesh, August 2005–July 2007

Surveillance site	Diarrhoea cases								Total
	Non-diarrhoea cases		Rotavirusdiarrhoea		Cholera		Non-rotavirus and non-cholera diarrhoea		
	No.	%	No.	%	No.	%	No.	%	
Bakerganj	163	63.7	31	12.1	27	10.5	35	13.7	256
Mathbaria	163	60.0	38	14.0	23	8.5	48	17.7	272
Total	326	61.7	69	13.1	50	9.5	83	15.7	528

Analysis of the overall dehydration status of the 457 under-five patients with acute watery diarrhoea observed by us revealed that 250 (55%) had no detectable signs of dehydration. One hundred and thirty-three (29%) children had signs of some dehydration, and severe dehydration was observed in 74 (16%) cases. Further analysis of the dehydration status revealed that most rotavirus-infected children had signs of some dehydration, which was significantly higher than cholera cases (47%; 54/114 vs 30%; 19/63; p<0.05) and ‘non-rotavirus and non-cholera’ diarrhoea cases (47%; 54/114 vs 21%; 60/280; p<0.001). In contrast, the proportion (51%; 32/63) of severe dehydration among the cholera patients was significantly (p<0.001) higher than among the rotavirus-infected patients (16%; 18/114) and ‘non-rotavirus and non-cholera’ diarrhoea patients (9%; 24/280). It was also noted that the combined proportion of severe dehydration of rotavirus and cholera accounted for 68% (50/74) of the total severe dehydration cases, which was significantly (p<0.001) higher than that of the ‘non-rotavirus and non-cholera’ diarrhoea patients (32%; 24/74).

Further analysis of severe dehydration status by age-group revealed that under-two children accounted for 83% (15/18) of severe dehydration among the rotavirus group (Table 3). This was significantly (p<0.01) higher than the proportion (31%; 10/32) observed among the cholera patients and was also higher (p<0.05) than the proportion (42%; 10/24) of the ‘non-rotavirus and non-cholera’ patients. Further, the highest proportion (44%; 8/18) of severely-dehydrated rotavirus-infected children was found in the age-group of 6-11 months. This age-group accounted for the largest proportion of rotavirus-positive patients.

**Table 3.** Distribution of severe dehydration by age-group among rotavirus, cholera, and ‘non-rotavirus and non-cholera’ cases in Bakerganj and Mathbaria, Bangladesh,   August 2005–July 2007

Age-group (months)	Rotavirus diarrhoea		Cholera cases		Non-rotavirus and non-cholera diarrhoea cases	
	No.	%	No.	%	No.	%
0-5	1	5.6	1	3.1	1	4.2
6-11	8	44.4	1	3.1	2	8.4
12-17	3	16.7	4	12.5	3	12.5
18-23	3	16.7	4	12.5	4	16.7
24-59	3	16.7	22	68.8	14	58.3
Total	18	100	32	100	24	100

All the 114 rotavirus-positive samples were ana-lyzed to determine the circulating rotavirus strains (G serotype and P genotype) in our rural surveillance sites. Analysis of strains revealed that the most frequently-identified strains were G1 P[8] (42%) and G2 P[4] (38%) (Table 4). These two strains accounted for about 80% of the identified circulating strains in our two surveillance sites. A number of other G and P strains were also identified, including G9 P[8] (4%) and G12 P[6] (4%).

**Table 4.** Distribution of G and P types of rotavirus strains from two surveillance sites: Bakerganj and Mathbaria, Bangladesh, August 2005–July 2007

Total no. of rotavirus strains	Typing of rotavirus completed	GP type	No. of rotavirus strain (%)				Total (n=114)		
			Bakerganj (n=45)		Mathbaria (n=69)				
			No.	%	No.	%		No.	%
114	114	G1 [P8]	14	31.1	34	49.3		48	42.1
		G1 [P6]	1	2.2	0	0		1	0.9
		G2 [P4]	17	37.8	26	37.7		43	37.7
		G2 [P6]	2	4.4	0	0		2	1.8
		G2 [P8]	1	2.2	0	0		1	0.9
		G2 [P0]	1	2.2	0	0		1	0.9
		G2 [P mixed]	1	2.2	3	4.3		4	3.5
		G4 [P8]	1	2.2	0	0		1	0.9
		G9 [P6]	0	0	1	1.4		1	0.9
		G9 [P8]	2	4.4	3	4.3		5	4.4
		G9 [P0]	1	2.2	0	0		1	0.9
		G12 [P6]	2	4.4	2	0		4	3.5
		G0 [P6]	1	2.2	0	0		1	0.9
		G0 [P8]	1	2.2	0	0		1	0.9

G2[P0], G9[P0], G0[P6], and G0[P8] were untypable

Analysis of the distribution of rotavirus-associated diarrhoea by months did not indicate distinct seasonal patterns in any of our surveillance sites compared to cholera, which showed clear seasonality. However, rotavirus was isolated almost throughout the year.

## DISCUSSION

Over the last few decades, remarkable improvements have been made in understanding the epidemiology, ecology, interventions, and prevention methods of diarrhoeal diseases. However, the incidence of diarrhoea appears to have remained stable globally (21). The present study was conducted in rural UHCs which are the grassroots-level rural health facilities that represent the mainstay for providing both outpatient and inpatient care for nearly 90% of the rural population of Bangladesh.

Our study has revealed that rotavirus infection was responsible for about 25% of the under-five children seeking treatment for acute watery diarrhoea at the rural health facilities. This was higher than observed previously (20%) in urban health facilities of Bangladesh (22). Our observation, however, is almost similar to that (27-36%) of the more recent study in rural Matlab (16). It should be noted that the ICDDR, B treatment centres in rural Matlab provide treatment exclusively for patients with diarrhoea and diarrhoea-associated illness. By contrast, the health facilities where our study was conducted provided treatment for all types of illness. Our study revealed that 77% of the rotavirus-infectedpatients were aged less than two years. Similar observations have been documented in a few other countries (23, 24).

In the recent decades, the proportion of hospitalizations due to diarrhoea attributable to rotavirus is thought to have increased (25). This study confirms the view that, in Bangladesh, diarrhoeal disease is one of the main causes of hospitalizations of children. In rural health facilities, children with dehydration, including those having signs of some dehydration, are usually hospitalized. We haveshown that, of all the under-five children hospitalized for various types of illness, 38% were admitted due to acute watery diarrhoea. This has not been previously documented in Bangladesh. We have also demonstrated that diarrhoea due to rotavirus accounted for 13% (range 12-14%) of all hospitalizations in under-five children in the two rural areas. This also, to our knowledge, has not beenpreviously reported in Bangladesh.

On a global scale, 500, 000-600, 000 infants and young children die from rotavirus-associated diarrhoea annually (4) due to limited access to healthcare facilities. Assessment of diarrhoeal deaths was not within the scope of this study. There were no deaths among the patients who attended our surveillance health facilities as prompt and adequate treatment was provided to them. It is known that diarrhoea patients with severe dehydration are at a higher risk of death if not promptly treated with intravenous fluid compared to patients having some dehydration, which can be corrected mostly with oral rehydration therapy.

A recent study in Matlab, Bangladesh, reported that 20% of rotavirus-infected children had signs of some dehydration, and only 0.3% had severe dehydration (16). This finding did not accord with the widely-accepted view that diarrhoea due to rotavirus is one of the major contributors to deaths in young children, particularly in developing countries. In contrast, our study revealed that over 47% of the rotavirus-affected children had signs of some dehydration, and about 16% were severely dehydrated. The findings of our study suggest that assessment of dehydration status based on pooled data of under-five children does not reflect the true proportion of age-specific severe dehydration; thus, the risk of death is involved. We have shown that the use of conventional grouping ‘aged less than five years’ revealed that 16% of rotavirus-infected children had signs of severe dehydration. This prompted us to look further into age-specific distribution of severe dehydration, which revealed that rotavirus-infected under-two children accounted for more than 80% of the severely-dehydrated patients. A study in Mainland China reported similar findings (26). The highest (44%) proportion of age-specific severe dehydration in our study was observed in children aged less than 6 months to 11 months. This observation contributes to the notion that children in this age-group are at a higher risk of death from diarrhoea due to rotavirus. The findings of our study suggest that rotavirus and cholera are the two major contributors to severe dehydration associated with acute watery diarrhoea; thus, the risk of death is involved, particularly in under-five children in rural areas of Bangladesh. It is known that enterotoxigenic *Escherichia coli* (ETEC) is also one of the major causes of acute watery diarrhoea, particularly in under-two children (27). However, as mentioned earlier, ETEC was beyond the scope of the current study.

In several countries, rotavirus tends to occur in cooler months (24). In Bangladesh, a higher incidence has been reported during the winter and monsoon months in the middle-belt regions (2). However, in our study sites, there were no distinct seasonal patterns of incidence of rotavirus. This may have been due to locations of our surveillance sites. Our study sites are situated in the southern coastal region of the country where the duration of cooler months is relatively much shorter, and the region is warmer than the middle-belt and northern areas of the country.

A study in Dhaka and Matlab, during 2005-2006, reported that G2 P[4] (43%) and G12 P[6] (11%) were the two most-prevalent circulating strains (2). By contrast, we observed that G1 P[8] (42%) and G2 P[4] (38%) were the most frequently-circulating strains in our surveillance sites in southern Bangladesh. The proportion of G12 P[6] strains found in our study sites was much lower (4%) than that observed in the earlier study (2). The difference in the distribution of serotypes isolated from different regions of the country suggests that circulating serotype within a country may have geographical variations.

Our study was limited to only one of the several geo-ecological regions of the country; therefore, this does not reflect the true magnitude of the disease in Bangladesh. However, we have demonstrated that about 25% of the under-five children seeking treatment for acute watery diarrhoea in rural health facilities were infected with rotavirus. Implication of this, on a national scale, will be significant given that acute watery diarrhoea is the leading cause of hospitalizations of under-five children in rural hospitals in Bangladesh (28).

Initiatives are underway for the introduction of rotavirus vaccine in countries where diarrhoea due to rotavirus is a public-health concern (29). However, efforts will be needed to improve the effectiveness of the vaccine and to use it in countries where it will be most beneficial. Ideally, these requirements can be achieved by implementing national surveillance systems for rotavirus which are efficient and reliable. Bangladesh qualifies on the priority list of countries for the introduction of rotavirus vaccine. Therefore, information-collection methods which will provide relatively reliable data, such as distribution of the disease in different geographical regions of the country, including seasonality and rate of hospitalization, need to be established.

## ACKNOWLEDGEMENTS

This research was funded by the Programme for Appropriate Technology in Health (PATH) (Grant No. GAV.1142-01-07231-LPS) and the US National Institutes of Health (NIH) (Grant No. 1R01A139129-01). ICDDR, B acknowledges with gratitude the commitment of PATH and NIH to the Centre's research efforts.

The abstract of the study was presented at the 12^th^ Asian Conference on Diarrhoeal Diseases and Nutrition (12^th^ ASCODD) held in Yogyakarta, Indonesia, from 25 to 27 May 2009.

Declaration of Interest: There was no conflict of interest.
